# Microdialysis coupled with droplet microfluidics and mass spectrometry for determination of neurotransmitters *in vivo* with high temporal resolution†

**DOI:** 10.1039/d4an00112e

**Published:** 2024-03-11

**Authors:** Shane S. Wells, Ian J. Bain, Alec C. Valenta, Ashley E. Lenhart, Daniel J. Steyer, Robert T. Kennedy

**Affiliations:** a Department of Chemistry, University of Michigan 930 N. University Ave Ann Arbor MI 48109-1055 USA rtkenn@umich.edu +1 734-615-4363

## Abstract

Monitoring the concentration fluctuations of neurotransmitters *in vivo* is valuable for elucidating the chemical signals that underlie brain functions. Microdialysis sampling is a widely used tool for monitoring neurochemicals *in vivo*. The volume requirements of most techniques that have been coupled to microdialysis, such as HPLC, result in fraction collection times of minutes, thus limiting the temporal resolution possible. Further the time of analysis can become long for cases where many fractions are collected. Previously we have used direct analysis of dialysate by low-flow electrospray ionization-tandem mass spectrometry (ESI-MS/MS) on a triple quadrupole mass spectrometer to monitor acetylcholine, glutamate, and γ-amino-butyric acid to achieve multiplexed *in vivo* monitoring with temporal resolution of seconds. Here, we have expanded this approach to adenosine, dopamine, and serotonin. The method achieved limits of detection down to 2 nM, enabling basal concentrations of all these compounds, except serotonin, to be measured *in vivo*. Comparative analysis with LC-MS/MS showed accurate results for all compounds except for glutamate, possibly due to interference for this compound *in vivo*. Pairing this analysis with droplet microfluidics yields 11 s temporal resolution and can generate dialysate fractions down to 3 nL at rates up to 3 fractions per s from a microdialysis probe. The system is applied to multiplexed monitoring of neurotransmitter dynamics in response to stimulation by 100 mM K^+^ and amphetamine. These applications demonstrate the suitability of the droplet ESI-MS/MS method for monitoring short-term dynamics of up to six neurotransmitters simultaneously.

## Abstract 2

Samples collected from brain of living subjects are segmented to nanoliter droplets and infused to the mass spectrometer. Temporal resolution for *in vivo* concentration dynamics of key neurotransmitters of 10 s is achieved. Created with Biorender.com.

## Graphical abstract

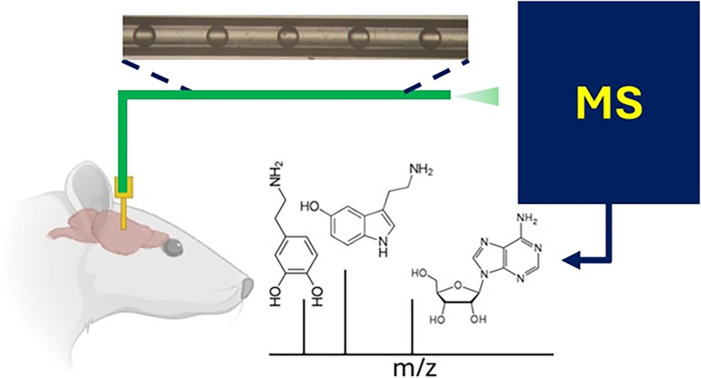

## Introduction

Intercellular chemical communication *via* neurotransmitters and neuromodulators is central to brain function. Monitoring extracellular neurochemical concentration dynamics in the brain of living subjects is valuable for understanding this chemical communication. Unlike measurements of neurotransmitter release *ex vivo* using tissues or cells, *in vivo* measurements allow monitoring with intact circuitry and correlation of behavior with neurochemical changes. Such measurements have played a role in understanding a wide variety of brain processes or diseases including learning,^1^ sleep,^2^ drug addiction,^3^ traumatic brain injury,^4^ Alzheimer's disease^5^ and amyotrophic lateral sclerosis.^6^

Multiple neurotransmitters may be signaling on short time scales in small brain regions and within a complex matrix of brain extracellular space. As a result, important metrics for *in vivo* measurements include temporal resolution, spatial resolution, selectivity, and multianalyte capability. Microsensors^7,8^ typically have excellent temporal resolution (<1 s has been achieved for a few analytes) and spatial resolution (some probes are in the micron size range); however, achieving adequate selectivity for the wide variety of neurotransmitters is challenging and multiplexing is limited. Sampling techniques such as microdialysis^9^ or push–pull perfusion^10–13^ typically have worse temporal resolution and spatial resolution, but when paired with appropriate analytical methods achieve excellent selectivity and multianalyte capability.^14–18^ Improvements in temporal and spatial resolution with sampling methods would broaden the scope of this approach and be valuable for neuroscience by providing a way to monitor multiple neurotransmitters *in vivo* at high temporal resolution.

Microdialysis sampling is performed using a ∼200–400 μm outer diameter (o.d.) by 1–4 mm long semi-permeable membrane probe implanted into the tissue of interest. The probe is perfused with a physiological buffer at 0.1–3 μL min^−1^ so that molecules below the molecular weight cutoff of the membrane diffuse into the sampling stream for collection at the probe outlet. Temporal resolution is limited by the requirement that enough analyte must be collected to exceed the limit of detection (LOD) of the accompanying analytical method. Using HPLC for analysis, the fraction collection interval required is typically >1 min. The relatively low throughput of HPLC often precludes operation at 1 min intervals so that 5–10 min temporal resolution is more typical since fewer fractions must be collected and analyzed. We and others have previously shown that it is possible to collect from sampling probes into nanoliter droplets, corresponding to seconds of fraction collection, that are separated by an immiscible carrier fluid.^19–29^ Droplet microfluidics facilitates manipulation of the small samples collected and interface to analytical methods with high-throughput and mass sensitivity like chip electrophoresis,^30,31^ electrospray ionization-tandem mass spectrometry (ESI-MS/MS),^32,33^ inductively coupled plasma-mass spectrometry (ICP-MS),^34^ electrochemical sensors,^22^ and luminescence assays^24^ to achieve temporal resolution of seconds.

ESI-MS/MS is particularly promising for analysis of *in vivo* samples given its versatility and potential applicability to most neurochemicals. Droplet ESI-MS/MS has previously been applied to simultaneous measurement of acetylcholine (ACh), glutamate (Glu), and γ-aminobutyric acid (GABA).^32^ In this work, we expand the method to include dopamine (DA), adenosine (Ado), and 5-hydroxytryptamine (5HT or serotonin) for multiplexed measurements at 11 s temporal resolution. In this work, low-volume microdialysis probes are interfaced to a 50 μm inner diameter (i.d.) cross junction to generate 5 nL dialysis fractions as droplets at 0.6 s intervals. At the cross, internal standard (IS) and diluent are added to the dialysate during droplet formation. Droplet trains containing over 1000 dialysate fractions are generated at the probe outlet and subsequently analyzed using ESI-MS/MS. The utility of the droplet fraction method is demonstrated by monitoring dynamics of neurotransmitters in response to brief administration of high K^+^ and amphetamine (AMPH).^35^ We also provide the first comparisons of direct ESI-MS/MS with HPLC-MS/MS to validate the concentration measurements made.

## Methods

### Reagents and materials

All chemicals and solvents were purchased from Sigma-Aldrich (St Louis, MO) unless stated otherwise. Perfluorodecalin (PFD) was purchased from Oakwood Chemical (Colombia Hwy, Estill, SC, USA). Isotopically labeled internal standards (d6-GABA, d4-ACh, ^13^C5-Glu, d4-DA, d4-5HT, d1-Ado) were purchased from CDN Isotopes (Quebec, Canada). For calibration, mixtures of all 6 standards were prepared in HPLC-grade water at 10 times the high point of the calibration curve (Table SI-1†), aliquoted, stored at −80 °C, and thawed for use daily as needed (single use). Mixtures of 200 nM internal standards were prepared in HPLC-grade water with 0.2% concentrated acetic acid, aliquoted, stored at −80 °C, and thawed for use as needed (single use). Artificial cerebral spinal fluid (aCSF) was prepared in 500 mL of water with 145 mM NaCl, 2.68 mM KCl, 1.4 mM CaCl_2_·2H_2_O, 1.01 mM MgSO_4_·7H_2_O, 1.55 mM Na_2_HPO_4_, and 0.45 mM NaH_2_PO_4_·H_2_O at pH 7.4. No phosphate (PO_4_) aCSF was prepared in 500 mL of water with 145 mM NaCl, 2.68 mM KCl, 1.4 mM CaCl_2_·2H_2_O, and 1.01 mM MgSO_4_·7H_2_O at pH 7.4. To mimic post-perfusion dialysate in standards, conventional aCSF and no PO_4_ aCSF were mixed 1 : 2 (33% PO_4_ aCSF). High K^+^ aCSF (100 mM KCl) was prepared as aCSF but with 47.68 mM NaCl and 100 mM KCl. d-Amphetamine hemisulfate was prepared in aCSF to a final concentration of 100 μM. The aCSF solutions were filtered through a sterile 0.22 μm polyethersulfone filter (Corning) and used for no more than 2 weeks.

### Droplet generation and transfer

For calibration, droplets were generated using a syringe pump to sip sample and carrier fluid alternately as described previously.^36–38^ Sipping was accomplished using a Harvard Apparatus PHD 2000 syringe pump fitted with a 25 μL gastight syringe (Hamilton, Reno, Nevada, USA) mated to a 20–30 cm length of 150 μm i.d. 360 μm outer diameter (o.d.) PFA tubing from IDEX (Lake Forest, Illinois, USA). Connections were made using low dead volume unions from Valco Instruments Co., Inc. (Houston, TX, USA). The open end of the tubing was moved from well to well of a 384-microwell plate using an XYZ-position manipulator while the pump was withdrawing at 700 nL min^−1^. The well plate had a layer of PFD carrier fluid over aqueous samples so that samples were segmented by PFD as tubing was moved between the two layers.

**Fig. 1:**
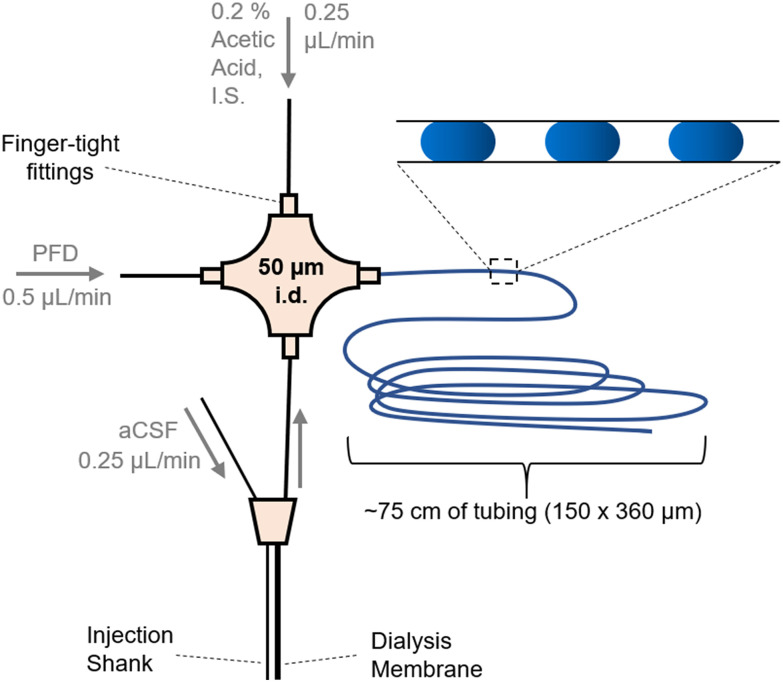
Layout for coupling a microdialysis sampling probe to droplet generation. Flows were in the direction of the arrows and driven by a syringe pump. Droplets (shown as blue ovals in the inset) were collected in tubing for later analysis by ESI-MS/MS. Injection shank was used to deliver stimulants (K^+^ or AMPH) to the vicinity of the sampling dialysis membrane. Fig. SI-1† has a photograph of the system.

When using a dialysis probe, droplets were generated by flowing PFD (carrier fluid), diluent (200 nM IS, 0.2% acetic acid), and dialysate into 3 arms of a 50 μm i.d. VICI cross junction. PFD was pumped at 0.5 μL min^−1^ while diluent and dialysate were both pumped at 0.25 μL min^−1^ using syringe pumps (IDEX, Middleboro, MA, USA) (Fig. 1 and Fig. SI-1†). Using this approach, dialysate emerging from the microdialysis probe was diluted 1 : 1 with incoming diluent and the resulting mixture was segmented into 3–8 nL droplets at 1–3 Hz and pumped into a 2–5 ft length of 150 μm i.d. × 360 μm o.d. PFA tubing. The resulting droplets were measured under a microscope to calculate average size of droplets in a train before MS analysis.

### Calibration curves

Calibration curves were obtained daily prior to collecting *in vivo* samples. Five droplets were generated for each concentration, and the middle three averaged for calibration. Signal from a droplet is obtained as the average of the points across a droplet.

### ESI-MS/MS

For time-resolved *in vivo* measurements. Tubing containing droplets were connected to nano ESI (nESI) emitters using zero-dead-volume Picoclear unions (New Objective, Woburn, MA). Emitters were pulled from 50 μm i.d. × 360 μm o.d. fused silica capillary to an i.d. of 15 μm and coated with conductive platinum (FS360-50-15-CE, New Objective, Woburn, MA). Direct nESI was performed by infusing droplet samples into a Micromass Quattro Ultima triple-quadrupole mass spectrometer (Waters, Milford, MA) at 50 nL min^−1^ with 1.4 kV ESI capillary voltage. Other nESI and MS/MS parameters are given in Tables S1 and S2.† MS/MS experiments were performed in multiple reaction monitoring (MRM) mode to scan for multiple mass transitions.

### Microdialysis probes

Custom side-by-side 2 mm long microdialysis probes were constructed for *in vitro* temporal resolution and *in vivo* sampling experiments. Inlet and outlet fused silica capillaries (40/110, i.d./o.d.) were glued together offset by 2 mm and inserted into a 4 mm piece of regenerated cellulose dialysis membrane (18 kDa MWCO, Spectrum Life Sciences LLC., Rancho Dominquez, CA). The distal membrane tip was sealed with an epoxy (Loctite, West Lake, OH) 100 μm from the inlet capillary. Dead volume within the membrane was eliminated by sealing the proximal end of the membrane around the inlet/outlet capillaries with epoxy and allowing it to wick into the membrane until it was within 100–200 μm of the probe active area. The probe was then secured within a 10 mm (25 G) piece of stainless-steel hypodermic sheath tubing (Small Parts Inc., Logansport, Inc.). A fused silica injection shank (75/150 i.d./o.d.) was added adjacent to the center of the membrane for 100 mM K^+^ or 100 μM AMPH injections. A custom designed 3D printed probe holder (VisiJet M3 Crystal, 3D Systems, Rock Hill, SC) was used to secure the probe. A 150/360 (i.d./o.d.) fused silica sheath was added to the outlet capillary to enable direct connection to the 50 μm i.d. cross junction for droplet generation.

### Microdialysis sampling

For *in vitro* recovery and temporal resolution experiments, microdialysis probes were inserted into a stirred vial of 2 mL of water maintained at 37 °C. To estimate system temporal resolution, a rapid concentration change was generated by spiking 10 μL of a 10 μM standard mix into the stirred vial (per 50 nM change). *In vivo* neurochemical measurements were performed in anesthetized male 25–30 g C57BL/6 mice (Envigo, Haslett, MI). Briefly, mice were anesthetized using 2–3% isoflurane and mounted to a stereotaxic instrument (David Kopf Instruments, Tajunga, CA). A 2 mm microdialysis probe was then implanted into the striatum using the following coordinate with respect to bregma: 0.6 mm anterior, ±1.75 mm lateral, and 4.2 mm ventral to the surface of the brain. Once lowered into place, probes were flushed continuously with aCSF for 15 min prior to droplet collection. As described above, droplets were generated directly from the outlet of the microdialysis probe. For neurotransmitter stimulation experiments, either 100 mM K^+^ aCSF or 100 μM AMPH solution were administered locally through the probe injection shank at 1 μL min^−1^ for 30 s, totaling 500 nL (Fig. 1). For potassium stimulation, 100 mM K^+^ aCSF was administered at 0 min and 5 min for a sampling period of 0–10 min. For AMPH stimulation, AMPH solution was administered at 1 min for a sampling period of 0–5 min. All procedures were approved by the Institutional Animal Care & Use Committee (IACUC) of the University of Michigan in accordance with Association for Assessment and Accreditation of Laboratory Animal Care (AAALAC) guidelines.

### Comparison of concentrations by ESI-MS/MS and LC-MS/MS

For some experiments, dialysis samples were collected and then split for analysis by both ESI-MS/MS and LC-MS/MS to validate the ESI-MS/MS concentrations measured. For these experiments, dialysis was performed in anesthetized 300–450 g Sprague Dawley rats (Charles River Laboratory, Wilmington, MA). Surgery was performed as described above with a cannula being implanted into the striatum using the following coordinates with respect to bregma: to bregma: 1.7 mm anterior, ±1.4 mm lateral, and 6.5 mm ventral to the surface of the brain. The animal was allowed to recover for two days before a 4 mm CMA12 microdialysis probe (Harvard Apparatus, Holliston, MA) was inserted into the cannula and microdialysis was performed. No PO_4_ aCSF was flowed continuously through the probe for 30 min at 2 μL min^−1^, then flowed at 0.5 μL min^−1^ for 30 min before samples were collected. Internal standard was added, then samples were split into two for separate analysis using LC-MS/MS and direct nESI-MS/MS.

For LC-MS/MS analysis, the fractions were subject to benzoyl chloride derivatization prior to analysis.^39^ For benzoylation, two parts sample are mixed with one part 100 mM sodium carbonate, one part 2% v/v benzoyl chloride in ACN, and one part internal standard in 1% sulfuric acid v/v in 20 : 80 MeOH/water added, step wise. The mixture was vortexed for 10 s after each addition. The internal standard mixture is comprised of analyte standards derivatized by the same procedure using ^13^C-benzoyl chloride as the derivatizing agent. Samples were analyzed using a Thermo Vanquish Flex UHPLC equipped with a Phenomenex Kinetex C18 chromatography column (150 × 2.1 mm, 1.7 μm, 100 Å) and interfaced to a Thermo TSQ Quantis triple quadrupole mass spectrometer. Mobile phase A and B consisted of water containing 10 mM ammonium formate/0.15% formic acid (v/v) and acetonitrile, respectively. The gradient used was: initial, 5% B; 0.01 min, 19% B; 0.68 min, 26% B, 1.05 min, 75% B; 1.8 min, 100% B; 2.18 min, 100% B; 2.28 min, 5% B; 3.00 min, 5% B at 600 μL min^−1^. The autosampler was kept at 10 °C, and the column was held at 55 °C, with 7.5 μL injection volumes.

For these comparisons, nESI-MS/MS analysis was performed on a Thermo TSQ Quantis triple quadrupole mass spectrometer. Emitters were pulled from 75 μm i.d. × 360 μm o.d. fused silica capillary to an i.d. of 5 μm and coated with gold using a sputter coater (Cressington Scientific Instruments, Watford, UK). Droplets were generated using a syringe pump as above. Samples were infused at 10 nL min^−1^ with 1.5 kV ESI capillary voltage.

## Results and discussion

### ESI-MS/MS parameters for neurotransmitter measurements

**Fig. 2:**
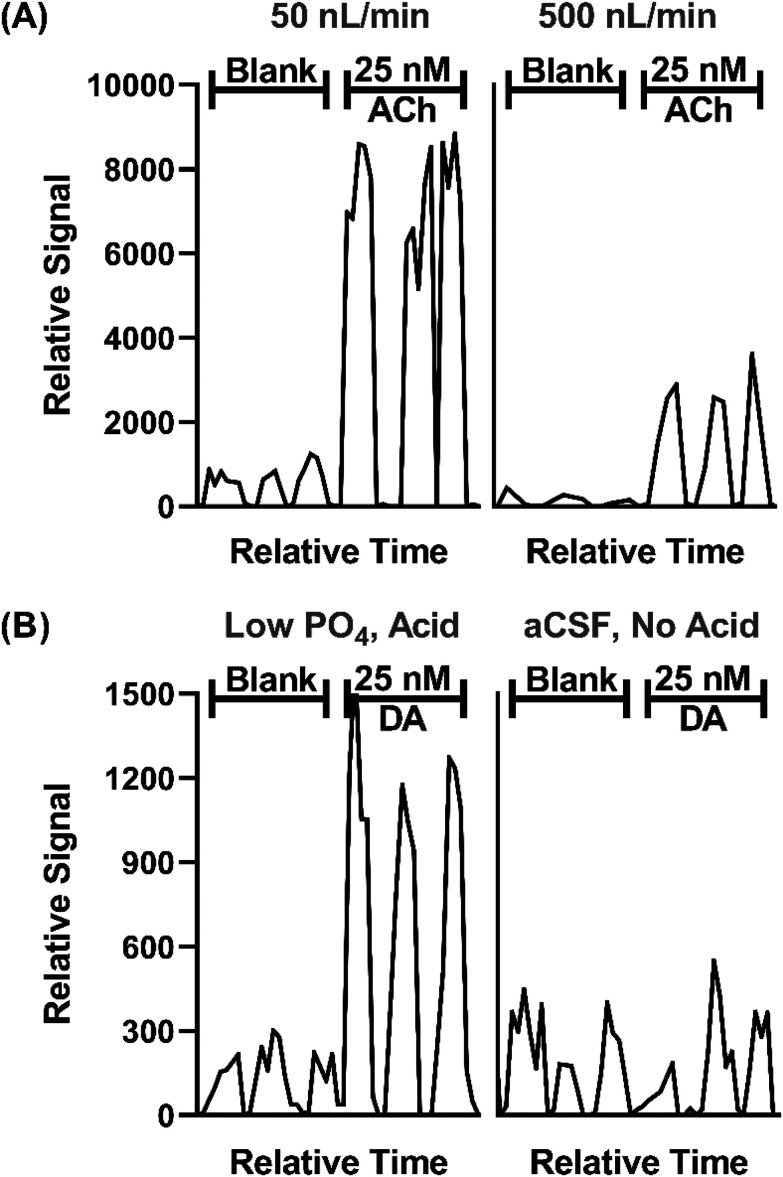
Effect of sample infusion rate (A) and background solution (B) on signal for neurotransmitters. All pertinent final conditions can be seen in Table SI-2.† (A) Comparison of MS/MS signals for background (blank) solution and 25 nM ACh at 50 and 500 nL min^−1^. Low infusion flow rates 15 μm emitter tip i.d. and 4.4 nL droplets. The higher flow rate used 30 μm i.d. tips and 40 nL droplets. Trace for three droplets for each conditions are shown. (B) An adjusted matrix with 33% of the standard PO_4_ concentration in aCSF along with a final concentration of 1% concentration acetic acid compared to standard aCSF with no acid. DA is shown for this comparison.

In prior work, we demonstrated that direct ESI-MS/MS on a triple quadrupole mass spectrometer of droplet samples could be used to detect ACh from microdialysis probes^33^ and ACh, Glu, and GABA collected from a push–pull perfusion probes.^32^ A key factor to achieve sensitivity was use of nanospray conditions consisting of low flow rates during infusion (<50 nL min^−1^) and small emitter tip diameter.^40–43^ Initial experiments for this project further support the significance of such conditions, as illustrated by the effect of flow rate and tip i.d. in Fig. 2A.

In this study we sought to expand the method to include DA, 5HT, and Ado while maintaining detection of ACh, Glu, and GABA. Initial studies showed that even with nESI-MS/MS, detection of the monoamine neurotransmitters (*i.e.*, DA and 5HT), which are present at low nanomolar concentration in various brain regions, was not feasible with conditions used previously. nESI and MS/MS parameters were tuned to improve LOD and signal-to-noise ratio (S/N) (Tables SI-1 and SI-2†). Adjustment of these parameters yielded modest LOD improvements; however, ultimately adjusting the aCSF composition was found to be an important modification. Although nESI reduces ionization suppression, we suspected that remaining ionization suppression from ions in the aCSF was hindering detection of the trace amines. To further improve analyte signal, aCSF was modified to contain 66% lower phosphate (PO_4_) content, an ion known to be a strong ionization suppressor, and 0.1% acetic acid was added to the sample, yielding a 4-fold increase in signal intensity and nearly half the noise for DA (Fig. 2B). The added acid presumably enhanced ionization and possibly helped stabilize DA as previous studies typically added acid to microdialysis samples for this purpose.^44,45^ Using the final conditions, summarized in Tables SI-1 and SI-2,† we found linear responses with detection limits of 2–36 nM for the 6 tested neurotransmitters dissolved in the modified aCSF (Fig. SI-1†). Subsequent work on more modern mass spectrometers with improved ion optics and electronics indicate that further improvements are feasible.

### *In vivo* selectivity

**Fig. 3:**
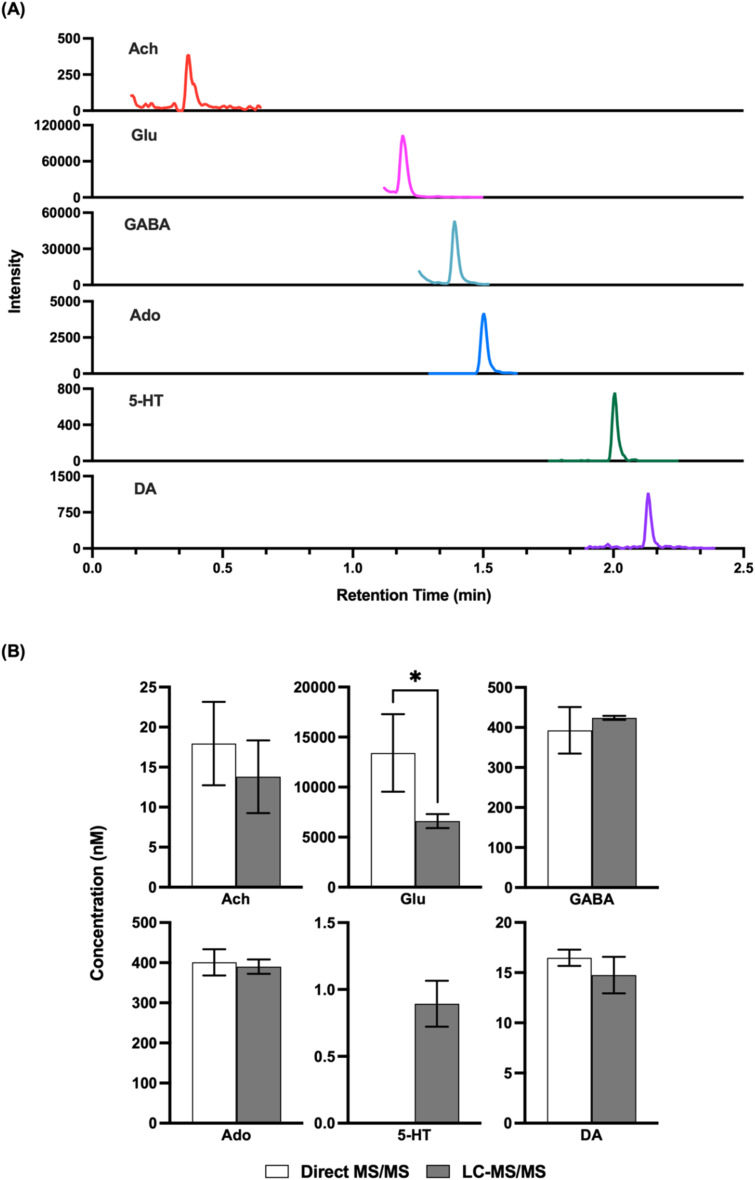
Comparison of direct analysis with MS/MS and analysis with LC-MS/MS. Dialysate fraction, as well as calibration samples, were split into two. One part was directly analyzed using MS/MS, while the other was derivatized using benzoyl chloride, and analyzed using LC-MS/MS. (A) Chromatogram of dialysate sample. (B) Bar graph comparing mean calculated concentrations from direct MS/MS and LC-MS/MS. 5-HT concentration was below detection limit for direct MS/MS. Error bars are +/− 1 standard deviation. Significant differences between the two groups were identified by Student's unpaired *t*-test and defined as *p* < 0.05 (*). For direct MS/MS *N* = 9, for LC-MS/MS *N* = 3.

Given that the LODs obtained appeared to be comparable to what would be needed for *in vivo* detection, we next attempted to record basal concentrations from *in vivo* samples and compare them to what was obtained by LC-MS/MS. Direct MS/MS is selective; however, with complex mixtures interference is possible and accuracy may be compromised by matrix effects, although the use of internal standards should account for this latter confound. Previous studies using direct MS analysis for neurotransmitters in dialysate have obtained basal concentrations that were comparable to that reported in other studies by other methods;^32,33^ however, given the variation in concentrations found in the literature we elected to do direct comparisons. As shown in Fig. 3, we found that analysis of the same fraction by direct MS/MS and LC-MS/MS yielded comparable concentrations for ACh, GABA, DA, and Ado. These results suggest that nESI-MS/MS has sufficient selectivity for these analytes in dialysate collected from the striatum The basal concentration of 5HT was below the LOD for direct MS/MS in these samples and therefore could not be compared. Glu did not yield a comparable result suggesting an interference in detecting this *m*/*z* transition by triple quadrupole MS. Further work, perhaps using a higher resolution mass spectrometer or different transitions, would be required to achieve selective measurement of Glu by direct MS.

### Droplet formation and temporal resolution

Temporal resolution of the droplet system was explored during *in vitro* experiments using the microdialysis probe to sample from a stirred vial. In microdialysis sampling, relative recovery (defined as concentration in dialysate over concentration in sample) is inversely related to dialysis flow rate.^46^ Given that the concentration of some of the neurotransmitters was near the LOD, we set the microdialysis flow rate to a relatively low 0.25 μL min^−1^ to achieve higher relative recovery than that obtained with the 1–2 μL min^−1^ often used. As in our previous study,^31^ relative recovery increased from 13–20% to 50–61% for different analytes with the flow rate decreased from 1 to 0.25 μL min^−1^. With this flow rate, we found that 5 nL droplets could be generated at ∼1.6 samples per s.

**Fig. 4:**
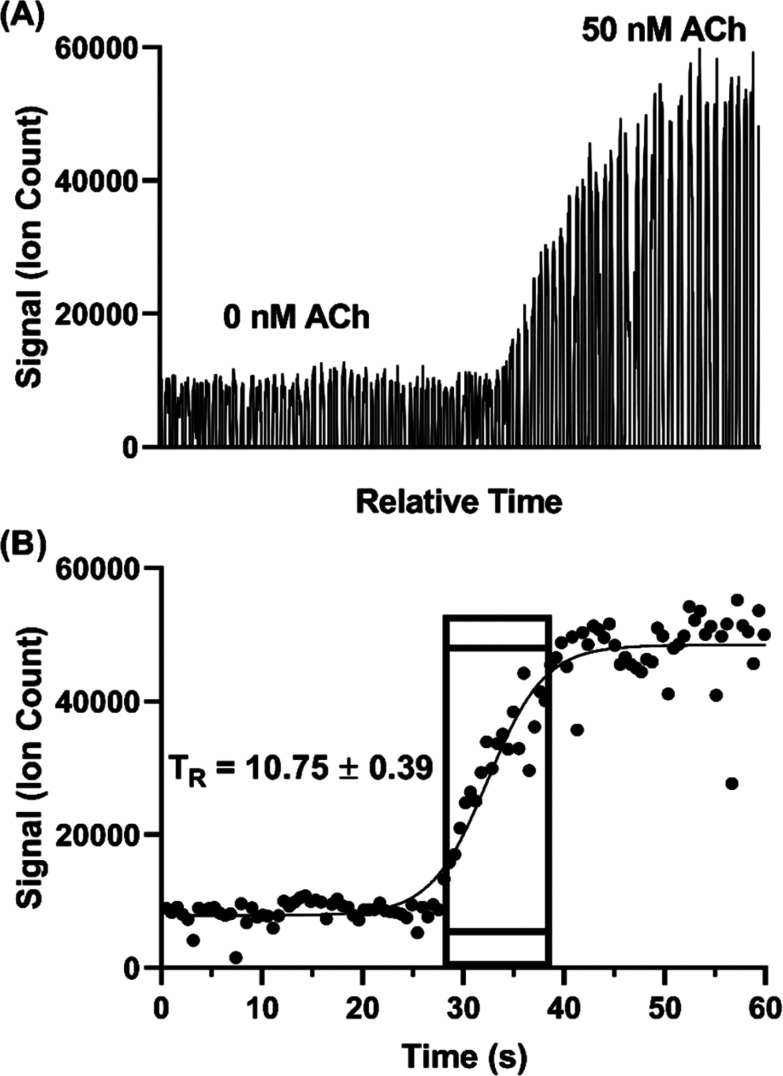
Measurement of temporal resolution of the microdialysis droplet sample collector. Temporal resolution measurements were performed by spiking ACh into a stirred vial to a final concentration of 50 nM while sampling by microdialysis at 250 nL min^−1^ with droplet sample collection. Resulting droplet samples were analyzed by ESI-MS/MS. (A) Extracted ion chromatogram of representative MS/MS measurement. (B) Temporal plot from measurement. Boxed area shows the range of 10% to 90% of maximal signal used to assess the response time. The signal was calculated as the average from the center points of each droplet. The time from the recording was correlated to real time based on the droplet volume/generation frequency (4.4 nL/1.89 Hz). The average temporal resolution (*T*_R_) and standard deviation of three replicates are reported in seconds.

Temporal resolution was evaluated by spiking standards into a stirred vial and monitoring the concentration change (*n* = 3). Fig. 4 illustrates a sample trace for ACh. The temporal resolution was calculated based on the number of droplets detected during the transition multiplied by the time taken to generate each droplet. The transition was defined as droplets between 10% above the average background signal intensity (*i.e.*, 0 nM ACh) and 90% of the average high concentration signal intensity (*i.e.*, 50 nM ACh,). Using this protocol, we found temporal resolution of 10.8 ± 0.4 s. This result is in good agreement with previous findings of about 11 s with the same flow rate through a 2 mm long probe.^31^ Notably, in the present work we used a commercially available, rather than custom microfabricated, cross. Thus, commercial fittings can be used without loss of temporal resolution.

Previous microdialysis with droplet collection methods for small molecules have achieved temporal resolution down to 2 s;^31^ but at sampling flow rates of 1.5 μL min^−1^. This better temporal resolution is due to the higher sampling flow rate, which washes out concentration pulses faster and in narrower bands. For this study, we favored the use of lower sampling flow rates with higher recovery to achieve adequate sensitivity for the low concentration amines. Temporal resolution could be improved by using higher flow rates if higher sensitivity in the measurement can be obtained *e.g.*, with newer mass spectrometers.

The probes used here, which had a 2 mm sampling length and 250 μm o.d., were of typical size for microdialysis; but the internal dimensions kept small as practical to enhance temporal resolution *i.e.*, larger internal volumes will give worse temporal resolution at a given flow rate. The probe size is a compromise as larger probes can increase recovery, but at the cost of worse spatial resolution and tissue damage. It may be also possible to design probes with better internal fluid dynamics to achieve high recovery and less temporal distortion.^28^

### Monitoring concentration dynamics *In vivo*

**Fig. 5:**
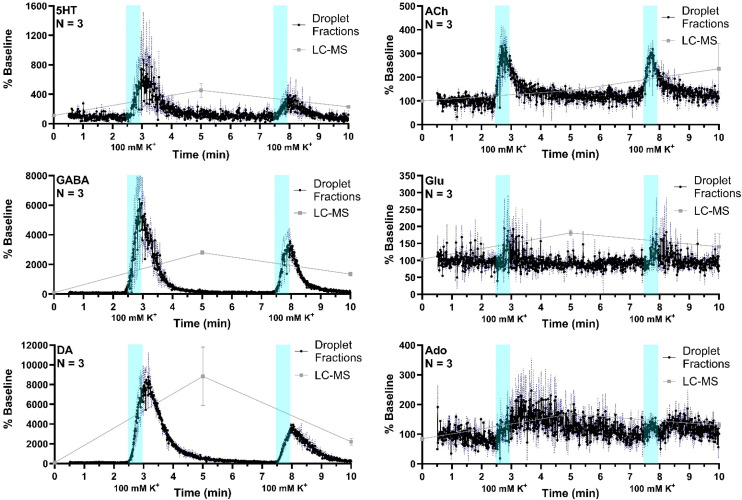
ESI-MS/MS recordings from *in vivo* experiments using microdialysis probes sampling the striatum over a 10 min. 500 nL of high potassium aCSF was administered over 30 s beginning at 0 and 5 min, with an approximate 2.5 min dead time indicated by blue shading. ESI-MS/MS signal from each droplet is converted to percent baseline (calculated by averaging signal from the first 60 s of recording) to normalize for concentration differences between animals. Black dots and connecting lines are signal and +/− 1 SEM is shown as a blue-dotted line (*n* = 3). Overlaid gray squares and connecting lines represent signals from LC fraction method, with fractions collected over 5 min intervals.

The system was then applied to record neurotransmitter concentration changes during K^+^ and AMPH stimulations. For high K^+^ administration, the striatum was sampled over 10 min, during which 500 nL of 100 mM KCl was administered (1 μL min^−1^ for 30 s) at two different time points, starting at 0 min and 5 min during sample collection, respectively, with a dead time of 2.5 min (*n* = 3). Droplet volume for the three replicates ranged from 5–6 nL. The average of all three replicates (expressed % baseline) are plotted with the standard error of the mean (blue-dotted line) for all compounds (Fig. 5). Data are plotted as the percent of baseline (calculated from the initial 60 s of data collected) to account for variations in recovery between experiments. Substantial increases are observed for 5HT, GABA, DA, and ACh in agreement prior results and expectations. Ado increased more modestly, somewhat obscured by the variability between droplets; but the changes are reasonable compared to other studies which showed ∼200% increase during 2 h 100 mM K^+^ infusion.^47^ Statistical significance of the increases for all of these compounds was *p* < 0.00001 using mixed-effects model to compare pre- and post-stimulation measurements. Glu showed a more ambiguous response that was not statistically significant (*p* = 0.52) in contrast to other reports of more robust of K^+^ evoked release in the striatum of rodents.^31,48^ This result may be because of the interference suggested by the selectivity experiments. It may also relate to the timing of *in vivo* measurement. For these stimulation experiments, fractions collection began within 15 min of probe insertion. It has previously been found that tissue disruption from probe insertion can affect responses for up to 16 h, therefore longer wait periods after insertion can provide more physiological responses.

To compare the difference in temporal resolution and monitoring dynamics between the droplet-ESI-MS/MS method and a conventional LC-MS/MS method, 5 min fractions were collected from the same mice and analyzed by LC-MS/MS. Though each method was used to analyze microdialysis fractions collected over 10 min, the LC fraction method provides only three time points which offers a temporal resolution of 5 min, while the droplet fraction method provides over 900 points with a temporal resolution of 10.8 s. This difference is clearly reflected in the neurotransmitter dynamics that can be seen in each trace. Though the LC fraction method shows similar percent baseline changes in the neurotransmitter concentrations, important temporal information is missing such as duration between stimulation and response, duration of elevated neurotransmitter concentrations, and rates of neurotransmitter release and reuptake/degradation. On the other hand, each of these details can be seen in the droplet fraction method, showing more suitability for monitoring short-term dynamics and rapid neurotransmitter changes.

**Fig. 6:**
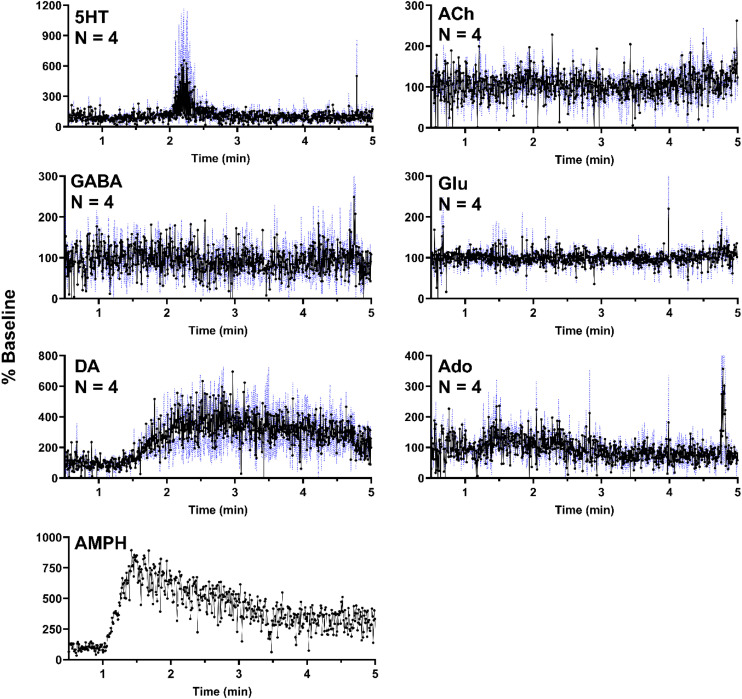
ESI-MS/MS recordings from *in vivo* experiments using microdialysis probes sampling the striatum over a 10 min with 500 nL of 100 μM AMPH administered over 30 s starting at 1 min. ESI-MS/MS signal from each droplet and for each analyte is converted to percent baseline (calculated by averaging signal from the first 60 s of recording) to normalize for concentration differences and all three traces were averaged (*n* = 4). Black dots and connecting lines represent droplet fraction method and +/− 1 SEM is shown as a blue-dotted line. AMPH was monitored for one of the four replicates.

In a separate set of experiments, the striatum was sampled over 5 min as 500 nL of 100 μM AMPH was administered locally (1 μL min^−1^ for 30 s). As expected, AMPH elicited a selective response, where significant changes were only present in DA (Fig. 6). The apparent but not statistically significant increase in 5HT was due to a single replicate which exhibited a substantial increase in a signal replicate (Fig. SI-2†) with the other replicates showing no response. This effect may be due to probe placement variations. AMPH has also been shown to increase extracellular 5HT.^49–51^ In addition to the six neurochemicals, AMPH in the extracellular space was also monitored in a single experiment, allowing for correlation between drug levels and neurotransmitter response (Fig. 6). Though DA is expected to increase in the presence of AMPH, we observe a delayed response, where DA concentrations began to increase approximately 30 s after AMPH appeared (Fig. 6). DA increased to 400% of basal levels within 30 s and remained elevated above baseline for an extended duration, decreasing to 300% baseline after three minutes. The extended increase in DA levels was most likely caused by the slow clearance of AMPH from the extracellular space (Fig. 6).

The variability or “noise” seen in the traces was caused by occasional sudden changes in signal between droplets. Although we cannot rule out that some of these fluctuations are due to endogenous fluctuations of neurotransmitter, it is more likely an artifact of the measurement. Such variability may arise from fluctuations in droplet size or composition (*e.g.* differences in the fraction of dialysate to internal standard added) during droplet formation in the cross or during transfer to the nESI emitter. Another possible source of fluctuations is inconsistent spray due to variability from the syringe pump with segmented flows. It is likely that such variability can be reduced with better engineering of the fluidics as other studies with other sample types have shown fewer such fluctuations.^37,43,52^

## Conclusions

Droplet fraction collection from microdialysis probes offers a way to achieve higher temporal resolution than standard approaches to microdialysis. In particular, droplet microfluidics enables manipulation of the nanoliter samples collected at seconds intervals. Using this capability however requires analytical methods capable of rapid, sensitive, and selective measurements on nanoliter droplets. In this work, we have demonstrated that ESI-MS/MS allows measurement of DA, Ado, and 5HT (at stimulated levels) beyond the measurement of ACh, Glu, and GABA previously demonstrated. The use of MS allows all compounds to be measured in each droplet for multiplexed monitoring with seconds temporal resolution. This capability extends what is possible for microdialysis sampling of multiple neurotransmitters. The 11 s temporal resolution allows more accurate assessment of rapid changes than typical microdialysis sampling and approaches that of some sensors; but, with multianalyte capability and selectivity of MS. This capability is envisioned to be of use in a variety of fundamental neuroscience investigations related to understanding the neurochemical correlates of brain functions. Higher temporal resolution is feasible with better mass spectrometers and fluidics. Also, this approach should be applicable to push–pull sampling devices for higher spatial resolution. The method required no microfabricated components and the assay required minimal sample manipulations making it relatively straightforward to use. The work also revealed limitations. Selectivity for Glu must be improved and sensitivity for 5HT is not yet sufficient for determining basal concentrations. Further work on these issues, as well as extending to other neurotransmitters will help increase the utility of this platform for neurochemical monitoring *in vivo*.

## Conflicts of interest

R. T. K. has equity interest in a company that seeks to commercialize droplet MS technology.

## Supplementary Material

AN-149-D4AN00112E-s001
