# Supplementary material for: Microdialysis coupled with droplet microfluidics and mass spectrometry for determination of neurotransmitters in vivo with high temporal resolution
Source: Analyst. 2024 Mar 11;149(8):2328–37. doi: 10.1039/d4an00112e (PMC11018092; doi:10.1039/d4an00112e)
Supplement: AN-149-D4AN00112E-s001 [file AN-149-D4AN00112E-s001.pdf]

Electronic Supplementary Material (ESI) for Analyst.
This journal is © The Royal Society of Chemistry 2024

Supporting Information for

Microdialysis Coupled with Droplet Microfluidics and Mass Spectrometry for Determination of Neurotransmitters *in vivo* with High Temporal Resolution

Shane S. Wells, Ian J. Bain, Alec C. Valenta, Ashley Lenhart, Daniel J. Steyer, Robert T. Kennedy*

Department of Chemistry, University of Michigan, 930 N. University Ave, Ann Arbor, MI 48109-1055

Corresponding Author:

Robert Kennedy

Email: rtkenn@umich.edu

Tel: 734-615-4363

Table SI-1: List of all transitions, dwell times, and collision voltages used for MS/MS analysis.

Compound	Parent (m/z)	Daughter (m/z)	Dwell (s)	Collision (V)	Calibration High Point (nM)	Calibration Low Point (nM)
**GABA**	104	87	0.15	10	200	10
**d6GABA**	110	93	0.03	12		
**AMPH**	136.1	119.1	0.03	15		
**ACh**	146	87	0.1	17	100	5
**Glu**	148	130	0.1	8	600	30
**d4ACh**	150	91.1	0.03	17		
**13C5Glu**	153	135	0.04	10		
**DA**	154	137	0.15	13	100	5
**d4DA**	158	141	0.05	13		
**5HT**	177	160.1	0.15	12	100	5
**d45HT**	181	164	0.03	15		
**Ado**	268	136.1	0.05	18	200	10
**d1Ado**	269	137	0.04	18		

Table SI-2: Summary of conditions used for ESI-MS/MS analysis of dialysate samples collected in vivo and for calibration.

nESI	Flow Rate	Capillary, Cone Voltage	Emitter i.d.	Drying Gas
	50 nL/min	1.4 kV, 35 V	15 µm	150 L/hr
Sample	Matrix (cal//tune)	Matrix (Perfusion)	Additive	Dilution
	aCSF (33% PO4)	aCSF (no PO4)	0.1% Acetic Acid	1:1, H2O
Analyzer	LM Res 1, 2	HM Res 1, 2	Ion Energy 1, 2	Exit, Entrance
	14.7, 14.5	14.5, 14.5	0.5, 0.4	5, -1 V
MS/MS	Span	Interscan Delay	Transitions/CE	Dwell Times
	0.3 m/z	2.5 ms	Table SI-2	Table SI-2

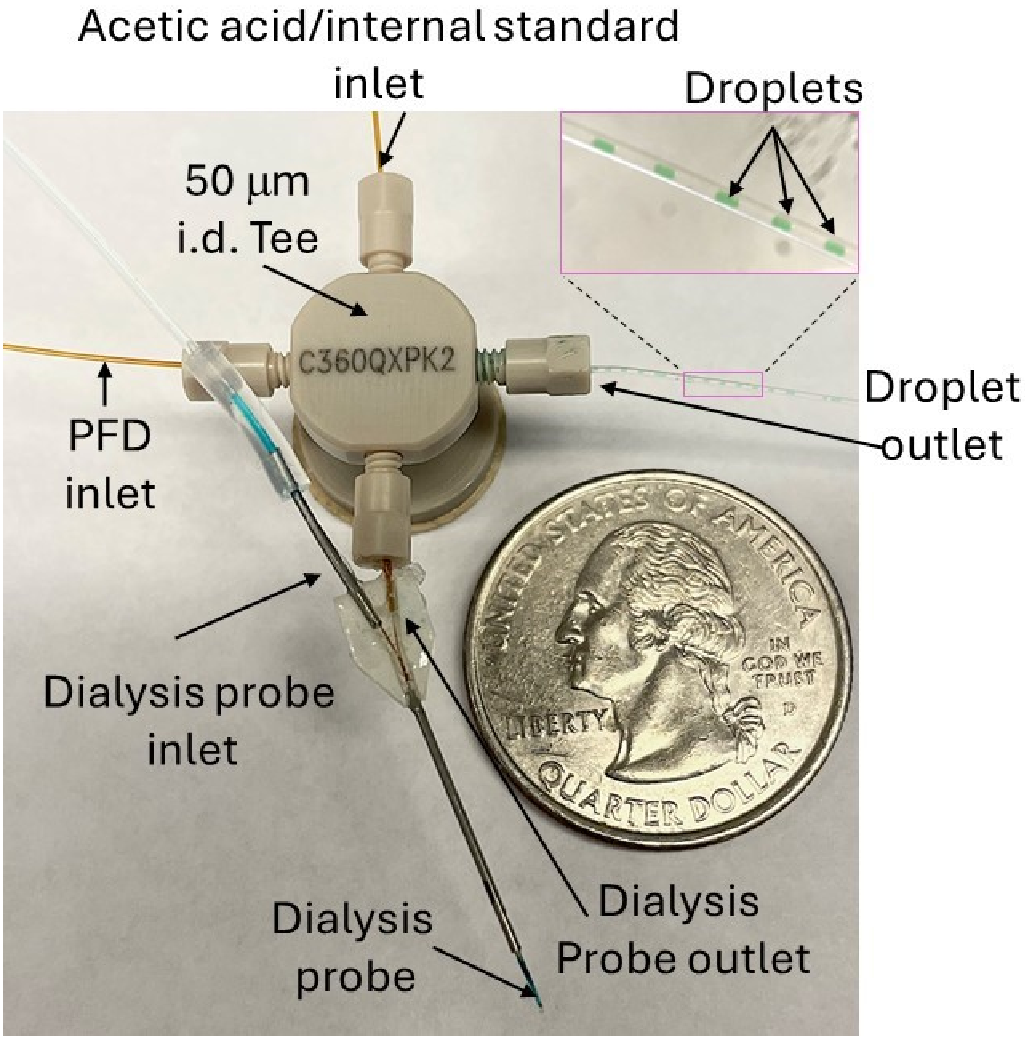

Figure SI-1. Photograph of probe and droplet formation system. Figure corresponds to drawing in Figure 1 in text, except this probe does not have an injection shank on it.

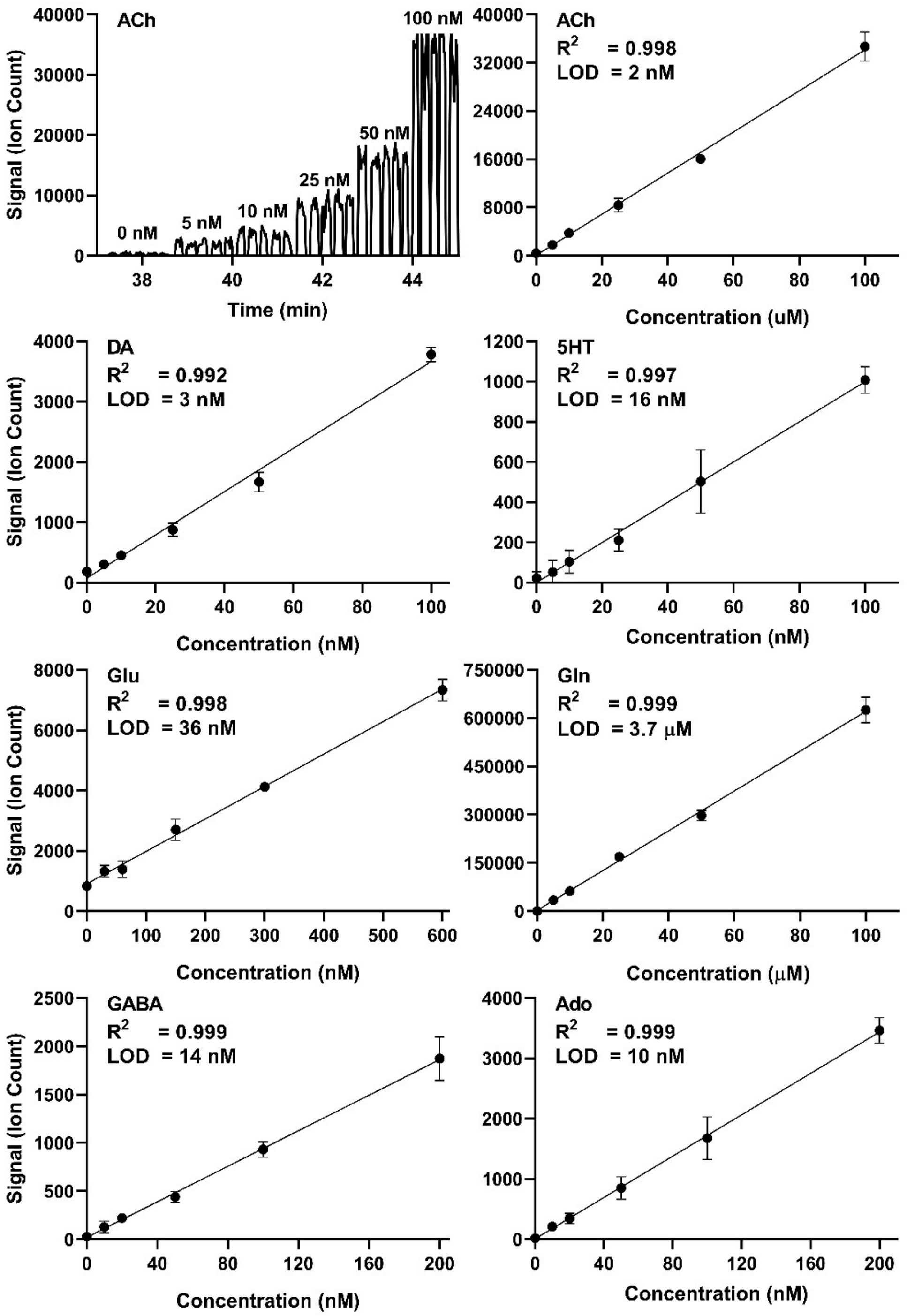

Figure SI-2. Calibration data for ESI-MS/MS of droplet samples of neurotransmitters. (Top Left) MS/MS trace of calibration data for ACh with 5 droplets for each of the 6 calibration levels. Samples were prepared from a well-plate and infused into the mass spectrometer as described in the text. (All other graphs) Average signal intensity versus standard concentration. Error bars are +/- 1 standard deviation for 3 replicates. These are the curves used for quantitation during one day of in vivo experiments. LOD is calculated using the limit of the blank method.

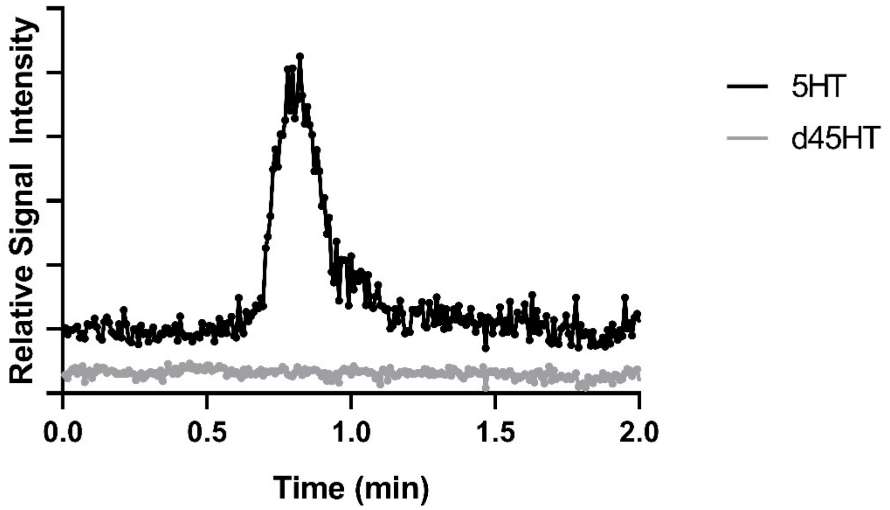

Figure SI-3. Example of single replicate that showed significant 5HT response to AMPH stimulation. The black dots and connecting lines represent MS/MS single for 5HT concentrations collected during AMPH stimulation and the gray dots and lines represent the internal standard d45HT used during droplet generation and nESI-MS/MS.
